# Early microglial activation in the TME enables FLASH-RT to eradicate medulloblastoma while promoting neuron-astrocyte crosstalk to minimize toxicity in the hippocampus

**DOI:** 10.64898/2026.03.16.712103

**Published:** 2026-03-18

**Authors:** Michèle Knol, Javier Franco-Pérez, Aymeric Almeida, Louis V. Kunz, Benoit Petit, Antoine Job, Jonathan Ollivier, Jackeline Romero, Jeannette Jansen, Veljko Grilj, Charles Limoli, Marie-Catherine Vozenin, Paola Ballesteros-Zebadúa

**Affiliations:** a LiRR - Laboratory of Innovation in Radiobiology Applied to Radiotherapy/Faculty of Medicine/University of Geneva, Geneva, Switzerland; b Radiotherapy and Radiobiology Sector, Radiation Therapy Service, University Hospital of Geneva, Geneva, Switzerland; c Laboratorio Patología Vascular Cerebral, Instituto Nacional de Neurología y Neurocirugía MVS, Mexico City, Mexico.; d Former affiliation: Laboratory of Radiation Oncology/Radiation Oncology Service/Department of Oncology/CHUV, Lausanne University Hospital and University of Lausanne, Lausanne, Switzerland.; e Institute of Radiation Physics (IRA)/CHUV, Lausanne University Hospital, Lausanne, Switzerland; f Department of Radiation Oncology, University of California, Irvine, CA. USA; g Laboratorio de Física Médica, Instituto Nacional de Neurología y Neurocirugía MVS, México City, Mexico.

**Keywords:** FLASH, Medulloblastoma, Microglial activation, Neuron-Astrocyte axis, Neuroinflammation

## Abstract

**Background.:**

FLASH-RT defines a promising treatment modality against medulloblastoma, as it minimizes treatment-related complications. To support its clinical translation, we dissected the cellular and molecular determinants of the FLASH response in the tumor-microenvironment (TME) and healthy hippocampus using an orthotopic human medulloblastoma mouse model treated with a hypo-fractionated FLASH regimen.

**Methods.:**

Five cohorts of 4 weeks-old UW228-MB-bearing female nude mice (n=57) were irradiated, or sham-irradiated using 3×10 Gy (BED=60), delivered 48h apart at 0.1 Gy/s (CONV) or 5.5×10^6^ Gy/s (FLASH) using an electron beam (eRT6). Digital spatial profiling (DSP) was performed 24h after radiotherapy in one cohort, while the four other cohorts were followed for long-term tumor response, cognition, and neuroinflammation.

**Results.:**

Both CONV and FLASH-RT induced a complete and long-lasting anti-tumor response in 100% of animals associated with cognitive decline. However, more mice maintained a very good discrimination score after FLASH exposure (38%) than CONV (7%). DSP revealed a sustained microglial activation in the cerebellar tumor micro-environment, where FLASH enhanced expression of genes with phagocytic and proteolytic activity. In the tumor free hippocampus, FLASH exposure induced a preferential neuron/astrocyte transcriptional crosstalk, which manifested over protracted times to minimize neuroinflammation and cognitive complications.

**Conclusion:**

The study shows the tumor-ablative efficacy of hypo-fractionated FLASH-RT in a human medulloblastoma mouse model. It is associated with qualitatively distinct transcriptional signatures prone to tumor and debris clearance mediated by microglial cells of the TME. Moreover, in the hippocampus, FLASH mitigates radiation-induced neurotoxicity by enhancing genes involved in synaptic plasticity, attenuating neuroinflammation, and preserving metabolic function.

## Introduction

The treatment of medulloblastoma (MB) is based upon biological and pathological medulloblastoma types, molecular biomarkers, staging, age at diagnosis and possible genetic predisposition. Multimodal treatment is composed of initial maximal safe surgical resection followed by craniospinal irradiation (CSI) of 23.4 Gy in 13 fractions, followed by a boost to the tumor bed (30.6 Gy in 17 fractions) for a total dose of 54 Gy, following the PNET 5 protocol (NCT02066220) for standard-risk medulloblastoma. Meanwhile, high-risk medulloblastoma patients receive CSI of 36 Gy in 20 fractions, with a boost (18 Gy in 10 fractions) for a total dose of 54 Gy and intensive chemotherapy regimens^1^. Sites of metastasis may receive additional RT boosts. While tumor control is excellent, with 80% of patients being cured, one major limitation of radiotherapy in children is the extreme vulnerability of the developing brain and the relative radiation resistance of brain tumors. The immature central nervous system is highly radio-sensitive, and the standard fractionation regimen used, composed of 1.8–2.0 Gy per fraction, is known to impair neurogenesis, synaptic development, and white matter maturation, ultimately leading to long-term neurocognitive deficits, including reduced IQ, memory impairment, and executive dysfunction^2, 3^. The survivors and their families therefore face challenges related to long-term side effects affecting their quality-of-life. The brain of young children is also known to have a lower tolerance to larger fraction sizes, limiting the possibility to apply altered fractionation regimen possibly more efficient against tumors. A last practical aspect is repeated anesthesia needed for the immobilization of the children during daily fractionated radiotherapy, introducing operational complexity both for health professionals and families, as well as medical risks associated with repeated sedation.

A recent meta-analysis showed that CSI using protons provides equivalent survival to photon-based regimen while significantly reducing growth hormone deficiency (GHD), hypothyroidism, mild ototoxicity, and neurocognitive toxicities in children with medulloblastoma^4^. However, novel therapeutic strategies able to further spare healthy brain and reduce treatment burden are needed. A decade ago, we pioneered FLASH radiotherapy^5, 6^, which can provide a unique opportunity to enhance therapeutic outcome by minimizing complications. In experimental adult and juvenile animal models, most studies using electron, photon and proton FLASH have demonstrated reduced cognitive decrements while maintaining anti-tumor efficacy^7–13^, even when hypo-fractionated regimen were used^14, 15^, except one^16^. Recently, the combination of a single 10 Gy dose of proton-FLASH with GD2-CAR-T cell therapy was shown to enhance survival of MB transgenic SmoM2 mice^17^.

The current study was designed to provide key missing elements to secure FLASH clinical translation for the treatment of children with brain tumors. A clinically relevant hypo-fractionated FLASH radiotherapy regimen (biologically effective dose (BED) of 60 Gy) achieved robust tumor ablation in an orthotopic human medulloblastoma model while mitigating radiation-associated brain injury. Cerebellar transcriptomic analyses revealed that microglial cells are the principal acute responders to irradiation, with FLASH promoting a metabolically active, phagocytosis-prone phenotype, in contrast to the proliferative and immunosuppressive signaling programs induced by CONV. In parallel, our findings also show how dose-rate may differentially shape long-term brain function as FLASH induced coordinated, circuit-specific neuron–astrocyte transcriptional programs rather than a uniform inflammatory response across three functionally linked hippocampal subregions known to be critical for memory encoding, pattern separation, and consolidation.

## Material and Methods

### Human medulloblastoma model in mice

**Figure 1.**
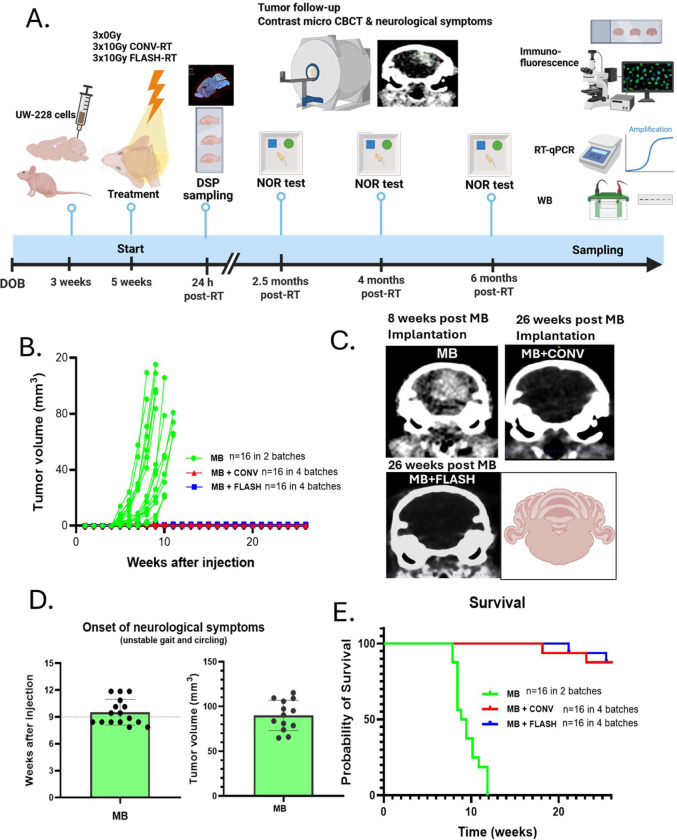
Long-term anti-MB response after FLASH-RT and CONV-RT. **A-** Diagram showing the timeline of the experiments. All animals were in good health at the time of behavioral studies and sampling was done in accordance with ethical procedures**. B-** Tumor volume was evaluated weekly for 26 weeks. **C-** Images obtained by micro-CT showing tumor growth 8 weeks after MB cell implantation and an isoefficient control of MB after FLASH-RT and CONV-RT. **D-** Control MB mice exhibited neurological symptoms between 9 and 12 weeks after injection, which was associated with a mean tumor volume of 90 mm^3^. **E-** Survival curves of MB-bearing mice after whole brain irradiation with a fractionation schedule of 3×10 Gy with FLASH-RT or CONV-RT.

Swiss nude mice (NU(Ico)-Foxn1^nu^) were bred in house and two-week-old females were used for all experiments. Five cohorts of animals were sequentially produced for a total of n=57 animals and included in the study (Fig. 1A). Experiment was approved by the Swiss (VD3603) Ethics Committee for Animal Experimentation and performed in accordance with institutional guidelines.

The UW228 cells were obtained from a 9-year-old female patient thanks to a donation from Prof. Martin Baumgartner, University Children’s Hospital Zürich, authorized by Dr. John Silber & Michael Bobola from Seattle Children’s Hospital and Regional Medical Center. UW228 human MB cells were cultured in a complete medium containing DMEM + GlutaMAX (31966–021, Gibco, USA) and supplemented with 10 % FBS (F7524, Sigma-Aldrich, USA). 1 million UW228 cells were engrafted in the cerebellum of juvenile mice right after the weaning (3 weeks of age), according to the following stereotactic coordinates: AP −6, ML +1, DV +2, at an angle of 30 degrees.

### Hypo-fractionated radiotherapy

Animals were divided into four experimental groups: non-irradiated tumor-free controls, non-irradiated tumor-bearing controls, and tumor-bearing mice treated with conventional radiotherapy (CONV), or FLASH radiotherapy. The radiotherapy regimen was composed of three fractions of 10 Gy delivered 48 h apart under anesthesia.

Mice were irradiated two weeks after tumor engraftment at 5 weeks of age. Whole-brain irradiation was delivered with 6 MeV Oriatron eRT6 electron beam linear accelerator (PMB, Alcen, France) using a graphite 17 mm half circle collimator, protecting the eyes but covering the entire brain, including the cerebellum, as previously described^10^, and using daily dosimetric control^18^. Each fraction of 10 Gy was delivered with CONV at 0.1 Gy/s or with FLASH in a single pulse at 5.5×10^6^ Gy/s.

### Tumor response

Tumor growth was monitored once per week using a micro-computed tomography (micro-CT) system at 55 kV, 0.19 mA, and 75 ms (MILabs, The Netherlands), using contrast enhancement (Accupaque 350 mg/mL; GE Healthcare AG, Switzerland). Tumor volumes were measured from DICOM files, performing slide-by-slide segmentation, and using an automated 3D model to calculate the final volume.

### Novel object recognition

The novel object recognition (NOR) test in mice is widely used to investigate radiation-induced cognitive decrements^19^ and has been performed as described previously in FLASH studies^8, 9, 20^. Statistical analyses were performed using GraphPad Prism (version 9.1). A Shapiro-Wilk test was applied to all groups to verify normal-distributed data. The discrimination index from the NOR test was compared using a one-way ANOVA and a Tukey multiple comparison test as post hoc analysis to determine the significance between all groups. The percentage of bad, good, and very good discriminators between CONV and FLASH was compared using the Z-score calculation for the two population proportions. For Z-score we used a free web tool (https://www.socscistatistics.com/tests/ztest/default2.aspx). Percentage of mice that were bad discriminators were defined in terms of exploration time of the new object of <60% of the time (below 18s, from a total of 30s of exploration time); good discriminators, were defined in terms of exploration time of the new object of >60% of the time (over 18s, from a total of 30s of exploration time), and very good discriminators were defined in terms of exploration time of the new object of >83.3% of the time (over 25s, from a total of 30s of exploration time).

### GeoMx Digital Spatial Profiling

To gain insight into the transcriptional response at an acute timepoint after the different irradiation modalities, spatial transcriptomic was performed on chosen brain areas. Regions-of-interest (ROI) were manually delineated in the cerebellum and in the dentate gyrus (DG), cornu ammonis 1 (CA1) and cornu ammonis 3 (CA3) of the hippocampi (Figure 3A). Two ROI were delineated in the cerebellum. Brains from 3 mice per group were sampled 24 hours post-irradiation (Figure 1A).

### Bioinformatics

Quality control, pre-processing, and further analysis of the data was performed in R (v4.4.0) as described in the supplementary material and method.

Differential gene expression (DGE) analysis was performed per region and cell type using a linear mixed model with the irradiation condition as a fixed effect and the slide as a random intercept to account for technical variability. The number of cell nuclei in each segment was counted to evaluate the number of cells analyzed in each segment. Gene expression in the DG was retrieved from an average of 30–150 astrocytes and 750–1100 neurons; in the CA3 from 30–80 astrocytes and 200–300 neurons; and in the CA1 from 15–100 astrocytes and 200–400 neurons. In the Cerebellum, gene expression was retrieved from an average of 30–60 microglial cells. Genes with an absolute log2 fold change >1 and a p-value of <0.05 were considered significantly differentially expressed. Gene set enrichment analysis (GSEA) was performed per region and cell type on gene lists ranked by the metric −(log2(fc)) · (−log10(*p value*)), combining the size of the effect and the significance. For further visualization, normalized expression values were used.

All scripts used for the analysis and plotting can be found at https://gitlab.unige.ch/lirr/dsp-pediatric-mb, as well as the raw sequencing data, which is available at https://www.ncbi.nlm.nih.gov/geo/ under accession number pending.

### Evaluation of neuroinflammation at endpoint.

Six mice of each group were sampled six months after radiotherapy by intracardiac perfusion with PBS, and the brain was processed to analyze neuroinflammation as described in the supplementary material and method.

## Results

### Both FLASH- and CONV-RT induce a complete and durable anti-MB response

48 juvenile mice were included and 32 underwent radiotherapy 2 weeks post-UW228 tumor engraftment. At this stage, tumors were detected by micro-CT imaging in 65% of the mice. RT induced complete and long-lasting response in 32/32 mice (Fig. 1B and C) transpiring in long-term survival (Fig. 1D) while 16/16 non-irradiated animals from 2 distinct cohorts developed MB visible on micro-CT imaging 8 weeks post-engraftment and developed neurological symptoms including unstable gating and circling within 9–12 weeks that required euthanasia (Fig. 1E).

### FLASH-RT preserves cognition better than CONV-RT

**Figure 2.**
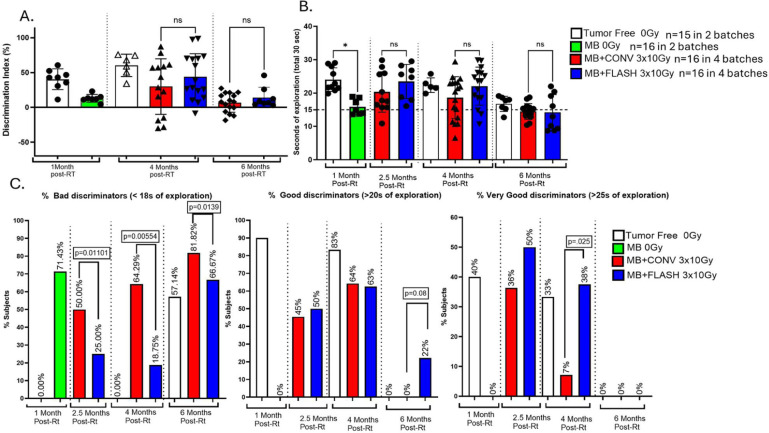
**A-** NOR test that was performed 2.5, 4, and 6 months after whole-brain irradiation with CONV-RT and FLASH-RT (3 × 10 Gy). Sham-irradiated tumor-bearing mice (3×0Gy) were only evaluated 1 month after irradiation as animals were sampled when ethical limit was reached due to tumor growth. Mice treated with CONV-RT appeared to have the lowest cognitive performance compared to the control and FLASH-RT groups. This was evident by two measures: the discrimination index evaluated during the first 5 minutes of video (a) and the fraction of time spent exploring the new object during the first 30 seconds of total object exploration (b). However, this trend did not achieve statistical significance. Data were compared using one-way ANOVA followed by the Tukey test for multiple comparisons. *p< 0.05, **p < 0.01. **B-** Then we measured the number of bad, good and very discriminators in each group. Bad discriminators were defined by an exploration time of the new object below 18s over a total of 30s of exploration time, good discriminators were by a time of exploration above 18s over a total of 30s of exploration time and very good discriminators were above 25s over a total of 30s of exploration time. (a) FLASH-irradiated groups had significantly fewer bad discriminators than CONV-irradiated groups 2.5-, 4-and 6-month post-RT while the percentage of good discriminators (b) was not statistically significant between FLASH and CONV (p = 0.08), but the percentage of very good discriminators was significantly higher FLASH-irradiated groups at 4-month post-RT only. Data were compared using the Z-score calculation for the two population proportions. *p< 0.05, **p < 0.01.

NOR tests were performed at various time points during the time course of the experiments to evaluate the impact of irradiation on memory. Age-matched tumor-free animals (n=10) were included in the analysis as control. Medulloblastoma-bearing non-irradiated mice were evaluated at 4 weeks, before neurological symptoms appeared. They showed alteration of the discrimination index (Fig. 2A) and were euthanized 8–12 weeks post-implantation upon progression of the medulloblastoma. Both irradiation modalities reduced the discrimination index and the novel object time of exploration when the test was performed 6 months post-RT (Fig. 2A, B). Tumor-free animals also showed a reduction in the novel object time of exploration at 6 months. Interestingly, when we selected mice according to their novel object time of exploration, significantly more bad discriminators were found in animals treated with CONV at all time points, whereas a higher proportion of very good discriminators were found in the FLASH-treated animals at 4 months (18% after FLASH vs 7% after CONV) (Fig. 2B).

### FLASH-induced transcriptomic response is region- and cell-specific

**Figure 3.**
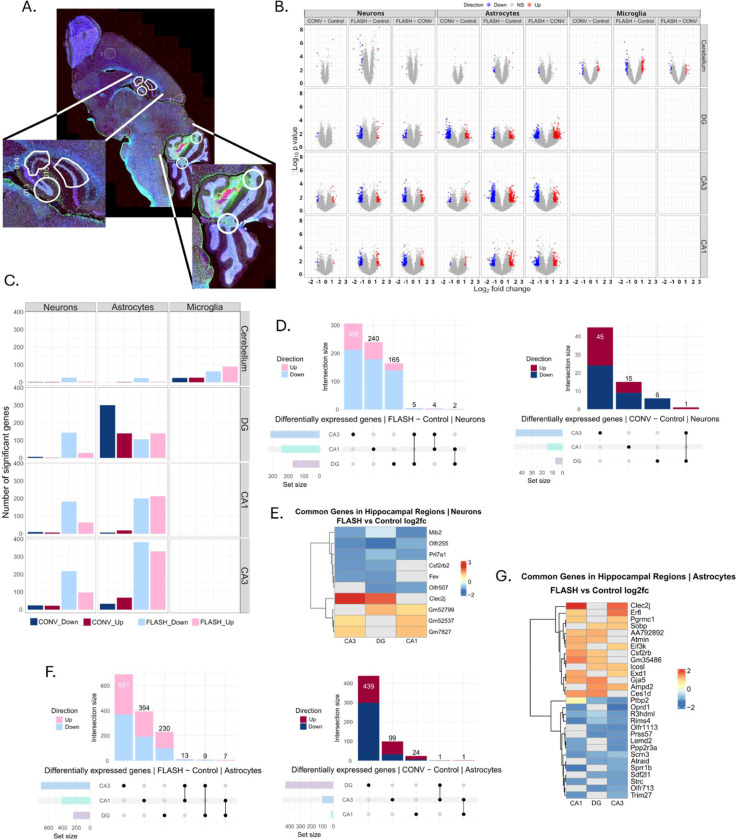
FLASH-induced transcriptomic response is region- and cell-specific. **A-** Representative image of a stained slide used for the DSP with the regions-of-interest in the cerebellum and the hippocampus (DG, CA1, and CA3). A 20x magnification was used. Nuclei are shown in blue, neurons in yellow, microglia in red, and astrocytes in green. **B-** Volcano plots showing the differential gene expression (DGE) analysis of all the regions and cell types. Cutoff values of |log2(fc)|>1 and p-value < 0.05 were used. **C-** Barplot of all significantly differentially expressed (DE) genes from the DGE, separated by region, cell type and irradiation modality. **D-** UpSet plots showing the intersections between the genes found significantly expressed in the different hippocampal regions in the neurons after FLASH (light blue and pink) and CONV (blue and red). **E-** UpSet plots showing the intersections between the genes found significantly expressed in the different hippocampal regions in the astrocytes after FLASH (light blue and pink) and CONV (blue and red).

Differential gene expression analysis in various regions of the brain (Fig. 3A) showed more transcriptional changes after FLASH than after CONV (Fig. 3B). Interestingly, in the cerebellum, only microglial transcriptomic was modulated by radiotherapy and differed between FLASH and CONV. Meanwhile, in the hippocampal regions, a radiation induced transcriptional profile was observed in astrocytes and neurons. We also found that FLASH induced specific transcriptional changes both in neurons and astrocytes in the DG, CA1 and CA3 subregions. These transcriptional changes in the neurons seemed to involve mostly downregulated genes, while in the astrocytes an equal proportion of down- and upregulated genes were found (Fig 3C). Contrary to that, CONV mainly altered the astrocytic transcription in the DG, while it had almost no transcriptional impact on the other regions or cell types at this timepoint (Fig. 3C). Furthermore, we observed that significantly differentially expressed genes were region-specific, while only a select few genes were common to multiple regions (Fig. 3D–E). Amongst those common genes was *Clec2j*, (C-type lectin like receptor 2), found to be upregulated after FLASH in the neurons of the CA3 and DG and the astrocytes of the CA1 and CA3 (Fig. 3E, G). Interestingly, *Clec2j* was also found to be upregulated in the neurons and astrocytes in the CA1 and CA3 after CONV. In the astrocytes of the CA1 and CA3 *Trh*, (tryrotropin-releasing hormone), was uniquely downregulated after CONV. Finally, *Mib2*, encoding a ubiquitin protein ligase, was downregulated after FLASH in the neurons of the CA1, CA3, and the DG (Fig. 3E).

### FLASH and CONV induce a distinct microglial transcriptomic imprint in the TME

**Figure 4.**
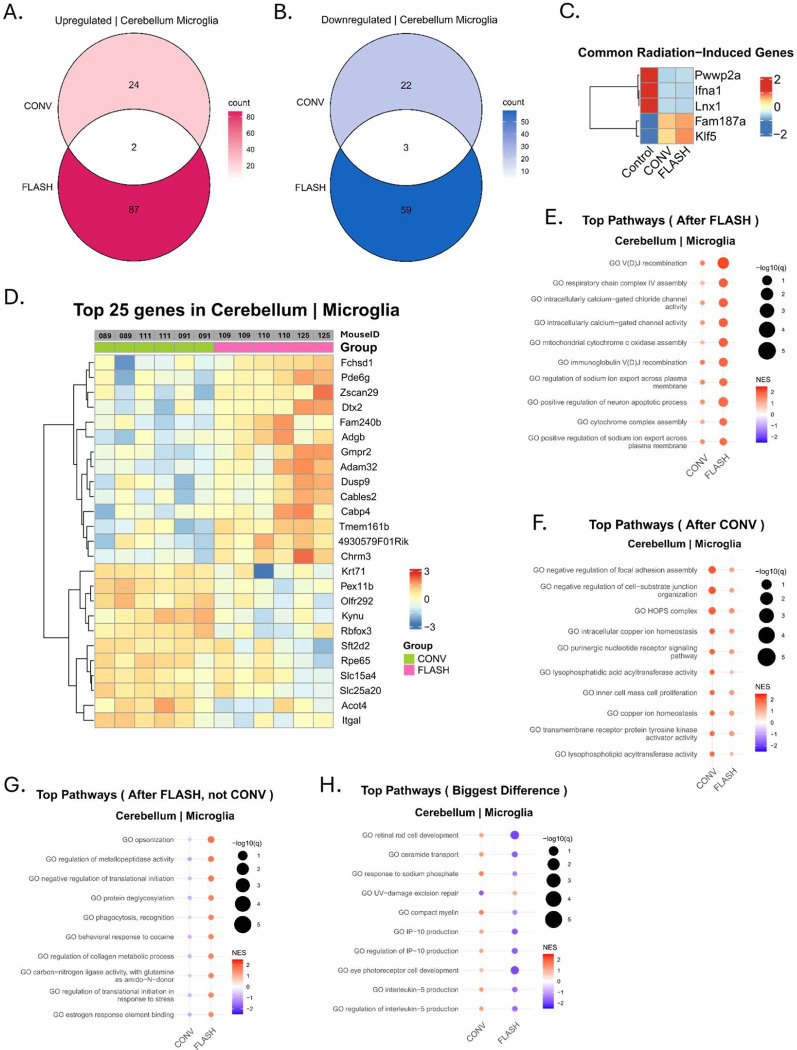
FLASH and CONV induce a distinct microglial transcriptomic imprint in the cerebellum. **A-** Venn diagram of the intersection between FLASH and CONV of the upregulated significant DE genes in the microglia in the cerebellum. **B-** Venn diagram of the intersection between FLASH and CONV of the downregulated significant DE genes in the microglia in the cerebellum **C-** Clustered heatmap of the common radiation-induced DE genes, from the intersections of the Venn diagrams, averaged per group and scaled per row. **D-** Heatmap with the top 25 DE genes with the biggest difference between CONV and FLASH. Genes were ranked according to |log2fc|*−log10(p-value). Columns represent a segment, two ROIs in the cerebellum were used per mouse. Rows were hierarchically clustered. **E-** Top 10 pathways from the GSEA ranked by the highest absolute normalized enrichment score (NES) after FLASH, where a positive NES (red) represents an upregulated pathway and a negative NES (blue) a downregulated pathway. The size of the dots shows the log10(q-value), i.e. the false discovery rate. **F-** Like E, pathways were ranked by the absolute NES after CONV. **G-** Similar to E, pathways were ranked according to the highest absolute NES after FLASH when NES after CONV was negative. **H-** Similar to E, pathways were ranked according to the biggest difference between the absolute NES after CONV and FLASH.

A microglial signature was identified to be the primary signal induced by irradiation in the cerebellum, while neurons and astrocytes were mostly silent (Fig 3B, C). A distinct profile was revealed after FLASH and CONV irradiation, where only 2 genes were commonly upregulated (*Fam187a* and *Klf5*) and 3 were downregulated (*Pwwp2a*, *Ifna1*, and *Lnx1*) (Fig. 4A, B, C). A total of 87 genes were found to be upregulated solely by FLASH, and 59 downregulated, meanwhile only 24 genes were uniquely upregulated by CONV and 22 downregulated (Fig. 4A, B, Supp. Table 2). The heatmap of the top 25 differentially expressed genes with the biggest difference between CONV and FLASH showed some variations in intensity within the different ROIs but nonetheless revealed a common imprint in the microglial cells, containing many newly identified, radiation induced genes (Fig. 4D). Additional GSEA analysis revealed that the FLASH-induced pathways are related to VDJ recombination, calcium/chloride gated channels and sodium ion export, as well as mitochondrial function (Fig 4E), suggesting a sustained microglial activity after FLASH exposure. Meanwhile, CONV-induced genes were mainly involved in iron related homeostasis and membrane/lipid biosynthesis, as well as promotion of proliferation and migration (Fig. 4F). The top enriched pathways uniquely induced after FLASH show enhanced phagocytic and proteolytic activity (*Megf10, Mmp16*) as well as tissue remodeling (*Adam19, Cd99l2, Spon2, Col13a1*), showing a microglial phenotype that is prone to ensure tissue clearance. Additionally, the top pathways showing the biggest difference in enrichment score between FLASH and CONV mainly revealed pathways upregulated by CONV and involved in immunomodulatory signals (promoting T cell infiltration *IP10/CXCL10, Il5*), ceramide, excision-repair and eye development (Fig. 4H).

### FLASH modulates the neuronal transcriptome in the hippocampus

**Figure 5.**
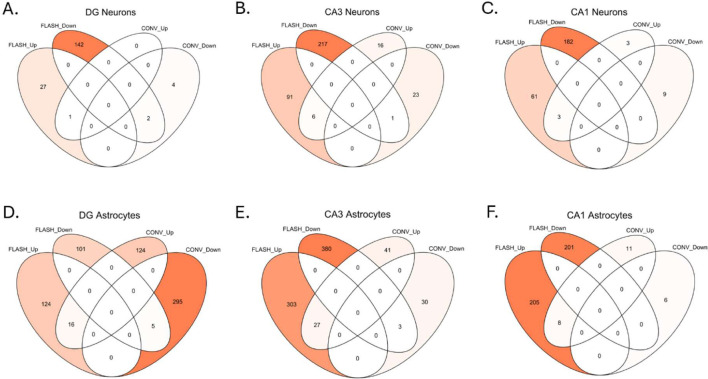
FLASH and CONV induce distinct neuronal and astrocytic transcriptomic imprints in the hippocampus. **A-** Double Venn diagram showing the intersections between the significantly up- and downregulated genes after FLASH and CONV in the neurons of the DG. **B-** Idem for the neurons in the CA3. **C-** Idem for the neurons in the CA1. **D-** Idem for the astrocytes in the DG. **E-** Idem for the astrocytes in the CA3. **F-** Idem for the astrocytes in the CA1.

Interestingly, radiation-induced signature in neurons was shown to be region-specific. In the DG, FLASH modified the neuronal transcriptome 24h post-irradiation, while the transcriptomic impact of CONV was minimal (Fig 5A). 28 upregulated and 144 downregulated genes were found after FLASH, with only 2 common downregulated genes and 1 upregulated gene between FLASH and CONV. These common genes were *Clec2j*, which was shown to be slightly more upregulated after FLASH compared to CONV, *H2*-*M1* and *Olfr995*, both downregulated after FLASH and CONV. We focused on the 28 upregulated genes found in the FLASH transcriptome (Supp table 2) and found many upregulated genes with unknown functions. However, we also found upregulation of genes involved in a coordinated stress-adaptive response involving DNA repair (*Ercc1, Bub1, Ube2d2b, Fbxo28),* neuroprotection by detoxification of lipid peroxidation products and oxidative stress mitigation (*Gsta4, Adh1, Pgrmc1),* mitochondrial regulation and proteostasis control (*Mrs2*, *Gpbar1)*. This early transcriptional response was accompanied by glial/immune activation (*Spib, Gpbar1)* and extracellular remodeling (*Eln, Egfem1*).

FLASH also modified the neuronal transcriptome in the CA3 (Fig 5B). 97 genes were found to be upregulated by FLASH, of which 6 were also upregulated by CONV: *Smcr8*, *Clec2j*, *Dock3*, *Emx1*, *Efna1*, and *Gm11554*. 218 genes were downregulated by FLASH, of which only 1 gene was shared between FLASH and CONV (*V1ra8)*. Meanwhile, CONV barely altered the transcriptome with only 16 upregulated and 23 downregulated genes. Similarly, focus on the 97-uregulated genes by FLASH (Supp Table 2) showed upregulation of genes important for activity-dependent plasticity axis involving *Arc, Camk2g, Adcy4, Calb1* amongst other genes *(Chrna4, Kcnip2, Adarb1, Pth2r, Pnoc)* known to regulate synaptic plasticity. As for the DG, genes involved in mitochondrial and metabolism were also found upregulated in the CA3 (*Dnm1l*, *Ckmt2,* Plin5), as well as genes related to axonal transport and vesicle trafficking (*Vps26b, Gak, Entr1, Bicd1, Bloc1s1, Golga5*) cytoskeleton remodeling (*Dock3, Arpin, Insc, Speg, Fras1, Adam20*), transcriptional reprogramming (*Emx1, Nkx1–2, Hoxb1, Klf3, Klf15, Zbtb9, Dpy30, Eid3, Cnot9*). No major inflammatory signal was found, but neuromodulatory genes were found to be upregulated (*Il1a, C1rl, Chil6, Vnn3, Hamp2*), suggesting that neuroinflammation is controlled. Overall, this pattern defines a preserved or enhanced CA3 neuronal function and maintained plasticity supported by active metabolism and controlled neuroinflammation.

Lastly, in the CA1, the transcriptional response of the neurons was again altered more after FLASH (Fig 5C). 64 genes were found to be upregulated by FLASH, of which 3 genes were upregulated by both FLASH and CONV (*Dsg1a*, *Dnajc21*, *Gm52537)*. 182 genes were downregulated by FLASH, and no genes were found to be shared between the two conditions. Meanwhile, only 3 genes were found to be upregulated and 9 genes to be downregulated by CONV. In the 64 upregulated genes by FLASH (Supp Table 2), many genes with unknown functions were found. Contrary to the “plasticity” genes found in CA3, this signature is more enriched in genes related to stress regulation such as *Atn1, Patz1, Ebf3, Bclaf3, Cdca7l, Ctdspl, Foxn1, Bsx.* Interestingly, the upregulation of *Esr1* suggest an estrogen-sensitive modulation of plasticity and a transcriptional control with *Aplf, Etaa1, Dzip3* consistent with a radiation-induced signature. *Ubqln1, Fbxo16, Fbxo30, Dzip3, Arrdc4* are related to proteostasis and *Akr1c6, Fabp6, Arrdc4, Etnppl, Apool, Mrpl3* to metabolic remodeling and lipid regulation. Several anti-inflammatory genes (*Entpd1*/*CD39*, *Cnr2)* as well as ion channels (*Asic4, Slc6a5, Slc30a2, Slc9b2*) are also worth including.

### FLASH and CONV induce a distinct transcriptomic imprint in the astrocytes in the hippocampus

Astrocytes in the three different regions of the hippocampus are not uniform, and as observed for the neurons in the hippocampus, radiation-induced transcriptome in the astrocytes was also found to be dose-rate dependent and region-specific. Interestingly, the ratio between the up- and downregulated genes in the astrocytes was similar, while in the neurons there were relatively more downregulated genes.

In the DG, both FLASH and CONV modified the astrocytic transcriptome 24h post-irradiation (Fig 5D). There were 140 genes upregulated by FLASH, of which 16 genes were also found to be upregulated after CONV. 106 genes were downregulated by FLASH, with 5 genes shared with CONV. Interestingly, CONV elicited the highest impact in the astrocytes of the DG, with 140 upregulated and 300 downregulated genes. We focused on the large upregulated astrocytic signature and found regulators of many astrocytic functions including cell cycle, chromatin protection and DNA surveillance programs (*Gadd45gip1, Hexim1, Atmin, Bid, Ezh2, Baz1a, Chrac1)* as well as soluble factors to promote neuron–glia communication (*Il33, Entpd3, Fgf2, Ffar2, Lcn2).*

In the CA3, there was a major transcriptional impact caused by FLASH (Fig 5E). 330 genes were found upregulated by FLASH, 27 were common between FLASH and CONV. 383 genes were downregulated by FLASH, with 3 common genes between FLASH and CONV. Meanwhile, only 68 genes were upregulated and 33 downregulated by CONV in this region. The upregulated genes also cluster into several coherent functional programs that suggest enhanced metabolic support (mitochondrial respiration: *Cox6a1, Cox8a, Ndufs6, Atp5a1*; antioxidant defense: *Sod2*; energy metabolism: *Pygb, Arg2*), synapse–glia communication (glutamatergic signaling: *Gria1, Grik4*; GABAergic signaling: *Gabarap, Glrb*; GPCR signaling: *Gpr158, Chrm4, Cnr2;* synaptic scaffolding/vesicle dynamics: *Bsn, Vamp2, Sv2b, Sh3gl2*), regulation of inflammatory signaling (chemokine/cytokine pathways: *Ccl2, Alox5*;Fc receptor signaling: *Fcgr1*; complement-related/immune regulators: *Cd300lb*), and chromatin remodeling consistent with transcriptional reprogramming (chromatin modifiers: *Hdac2, Ash2l, Rnf2*; and transcription factors: *Elk4, Fosl1, Hey1*).

In the CA1, the DSP again showed that FLASH modified the astrocytic transcriptome more than CONV, albeit slightly less than was found in the CA3 (Fig 5F). 213 genes were found upregulated by FLASH, 8 were common between FLASH and CONV. 201 genes were downregulated by FLASH, with no common genes with CONV, which by itself upregulated 19 genes and downregulated 6 genes in this region. The upregulated genes show a prominent signature in ECM production and remodeling (collagens/ECM components: *Col1a1, Mxra7*; cell adhesion molecules: *Ctnna1, Itgb5, Gja5* and tight junctions: *Cldn22*) and suggest a reactive phenotype of the astrocytes (innate immune receptors: *Tlr4, Nlrc5*; interferon/cytokine signaling: *Ifng, Ifngr2, Csf2rb*; chemokine receptor: *Cxcr3*). Furthermore, they are involved in steroid/lipid metabolism (cholesterol/steroid enzymes: *Hsd3b7, Hsd3b3*; ABC transporters: *Abca9* ; nuclear receptors: *Esrra, Vdr*), transcriptional control (histone modification enzymes: *Kdm5b*; developmental transcription factors: *Hoxa1, Foxo6, Hey2*; mediator complex: *Med21)*, and synaptic modulation (GABAergic signaling: *Gabra1*; potassium channels: *Kcnk5, Kcng4;* TRP channels: *Trpc3*; GPCR signaling: *Adcy1, Gnas*) (Supp. Table 2).

### FLASH reduces neuroinflammation and glial activation at protracted times

**Figure 6.**
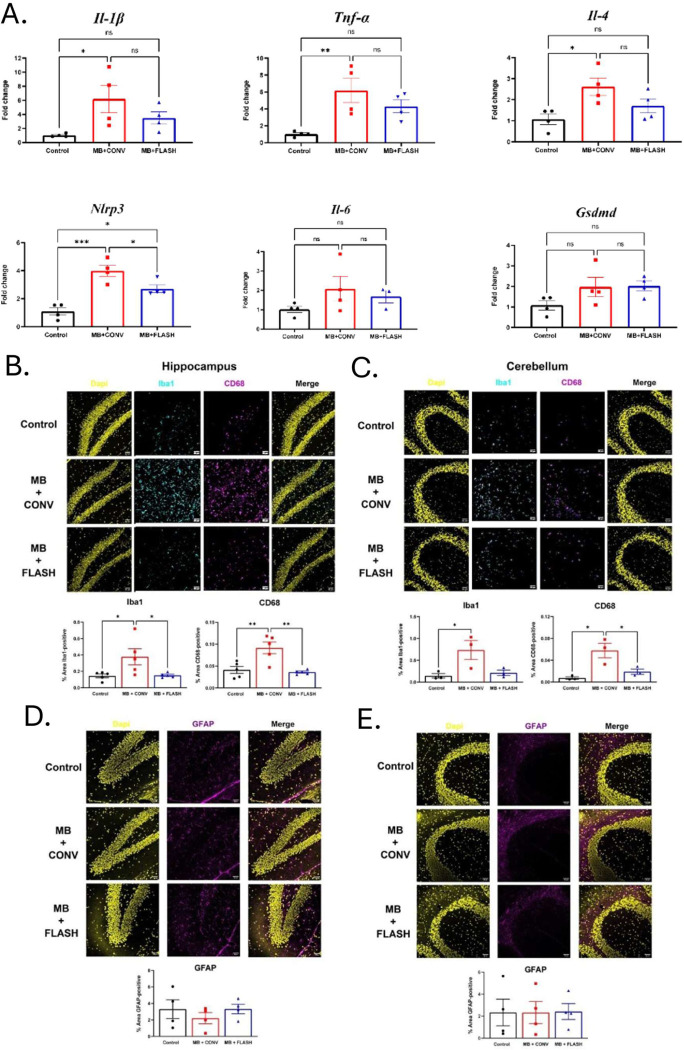
Neuroinflammatory status 6 months post-irradiation. **A-** Gene expression of the inflammatory markers *Il-1b, Tnf-a, Il-6, Nlrp3, Il-4* and *Gsdmd* in the whole brain of control and mice treated with CONV-RT and FLASH-RT. Data are expressed as mean ± SEM (n =4 per group). The analysis was done with one-way ANOVA and Tukey’s post hoc test. *p< 0.05, **p < 0.01, ***p < 0.001. FLASH-RT spares microglial activation in the hippocampus and cerebellum. **B, C-** Immunofluorescence staining of Iba1 and CD68 in the hippocampus (B) and the cerebellum (C) increased after CONV-RT but not in the mice treated with FLASH-RT. **D, E-** No significant changes were observed in the hippocampus (D) and cerebellum (E) when we analyzed GFAP as an astrogliosis marker. Data are expressed as mean ± SEM (n = 3–4 per group). The analysis was done with one-way ANOVA and Tukey’s post hoc test. *p< 0.05, **p < 0.01, ***p < 0.001. Scale bar: 50 me.

At endpoint (6 months post-irradiation), age-matched tumor-free non-irradiated animals and irradiated animals were sampled to investigate neuroinflammatory status and glial activation in the cerebellum and the hippocampus. A panel of pro-inflammatory cytokines was analyzed by qRT-PCR on whole brain slices. *Il-1β, TNF- α*, IL4 and *Nlrp3* mRNA levels were found to be enhanced uniquely after CONV irradiation vs control, while the level of IL-6 and Gasdermin-d mRNA was not different in control and irradiated animals (Figure 6A). Iba1 and CD68 slides stainings were also used to monitor glial activation. Both in the cerebellum and the hippocampus, high expression levels of *Iba1* and *CD68* was measured in CONV-treated animals, whereas glial activation was low and similar in FLASH-treated and tumor-free non-treated animals (Figure 6C, D). No astrogliosis monitored by GFAP staining was found in the irradiated animals as compared to non-irradiated animals (Figure 6E, F).

## Discussion

Hypo-fractionated FLASH radiotherapy delivered in 3 fractions of 10Gy, achieved complete and durable control of medulloblastoma compared to conventional dose-rate irradiation. The delivered BED (60Gy, α/β=10) was equivalent to standard of care. Early radiation-induced cell-specific transcriptional signatures were generated 24h after radiotherapy across multiple regions-of-interest including the cerebellum, where the MB cells were engrafted, and in tumor-free hippocampal subregions. The signatures show that radiotherapy triggers distinct cerebellar microglia differentiation. However, while FLASH induces genes prone to phagocytic and proteolytic activity in microglial cells, CONV preferentially upregulates reactive genes prone to immunomodulation, iron homeostasis, membrane and lipid biosynthesis and proliferation. Simultaneously, the FLASH-induced signatures across hippocampal subregions revealed a coordinated yet region-specific transcriptional reprogramming of both neurons and astrocytes along the tri-synaptic circuit. This signature revealed enhanced synaptic plasticity, reduced neuroinflammation, and preserved metabolic function. Each of these primary pathways participates in the mitigation of cognitive decline and decreased chronic neuroinflammation as compared to CONV.

The cerebellar signature revealed highly divergent transcriptional programs in microglial cells after FLASH vs CONV, highlighting minimal overlap between the two modalities. FLASH triggered a markedly broader transcriptional reprogramming, with 87 genes uniquely upregulated supporting sustained metabolic activation and ionic remodeling consistent with prolonged microglial engagement. The enhanced phagocytic and proteolytic activity is indicative of a microglial phenotype oriented toward debris clearance and tissue remodeling. In contrast, CONV preferentially upregulated pathways related to iron homeostasis, membrane and lipid biosynthesis, and gene programs supporting proliferation and migration. While our study has been performed in adaptive immunocompromised animals, the myeloid contribution remained intact, and our findings are consistent with two recent reports^17, 21^ showing a differential role for the microglial cells after FLASH vs CONV. In diffuse midline glioma, Padilla et al. showed that electron irradiation in both FLASH and CONV were able to activate an IFN1 response, while early activation of microglial cells occurred uniquely after FLASH, as is the case in this present study. In a transgenic medulloblastoma (*Math1-Cre/smoM2*^+/−^) model, Ni et al. used proton FLASH irradiation showing that it abrogates lipid oxidase expression and oxidized low-density lipid generation to reduce PPARγ activity. Meanwhile, CONV induced reactive oxygen species-dependent PPARγ, resulting in differential and immunosuppressive polarization of tumor-infiltrating macrophages. Interestingly, in our study, the pathways exhibiting the greatest differential enrichment between CONV and FLASH were predominantly CONV-driven. They included proliferative and immunomodulatory signaling cascades dependent upon IP10- and IL5-pathways after CONV while FLASH promoted a metabolically active, phagocytosis-prone state potentially supportive of efficient tissue clearance, able to shape a distinct inflammatory milieu. However, in this model both modalities ultimately lead to equivalent and complete tumor response, suggesting that in both cases the high dose regimen selected drove the therapeutic outcome and was ablative.

The hippocampal signatures were dissected from three ROIs located in functionally linked and sequential areas: the DG, essential for memory encoding; the presynaptic CA3, essential for pattern separation; and the post-synaptic CA1, essential for pattern completion ^22, 23^. The hippocampal signatures were distinct in these three regions and revealed that FLASH was able to modify the transcriptional programs of the neurons and astrocytes at an early time point. Strikingly, the FLASH regulated genes found in astrocytes align closely with the pattern of each region to support preserved neuronal functions ^24^. In the DG, astrocytes help to maintain firing required for pattern separation ^25^, in the CA3, they have been shown to protect neurons against ischemia by suppressing intracellular Ca^2+^ overload ^26^ and in the CA1, the astrocytes regulate synaptic plasticity and information integration ^27^.

In the neurons of the DG, FLASH-induced upregulation was enriched for genes supporting cell cycle–associated chromatin dynamics, DNA repair, mitochondrial function, and metabolic adaptation, consistent with heightened structural plasticity and activity-dependent remodeling. Mitochondrial and metabolic function seemed sustained with upregulation of *Dnm1l,* known to control mitochondrial fission^28^; *Ckmt2,* which is a mitochondrial creatine kinase required to maintain energy homeostasis^29^, and *Plin5,* known to decrease the exposure of mitochondria to fatty acid by storing them in lipid droplets ^30^, the latest also found upregulated in the CA3. Concomitantly, DG astrocytes exhibited signatures related to metabolic support, lipid handling, and extracellular matrix modulation, suggesting enhanced neuron–glia metabolic coupling to sustain plasticity as proposed previously ^31, 32^. In addition, the up-regulation of *Gadd45gip1, Hexim1, Atmin, Bid, Ezh2, Baz1a, Chrac1* after FLASH exposure, suggest that FLASH-exposed astrocytes are activating cell cycle control, chromatin protection and DNA surveillance programs but not overt pro-apoptotic collapse prone to maintain tissue stability. The upregulation of *Il33, Entpd3, Fgf2, Ffar2, Lcn2* also suggests active neuron–glia communication (*Il33)*, fine-tuned immune tone (*Entpd3)* rather than overt inflammation occurs and trophic support signaling with upregulation of *Fgf2*.

In the CA3, FLASH modulated neuronal transcripts prominently reflect synaptic organization, vesicle trafficking, and excitatory transmission, while CONV induced only modest changes. The neuronal signature prominently involved the activity-dependent plasticity axis, including *Arc* ^33^, *Camk2g* ^34^, *Adcy4* ^35^, *and Calb1* ^36^ supporting preserved synaptic plasticity. This aligns with the pattern-separation properties of the CA3 and is consistent with the synaptic vesicle recycling and long-term potentiation (LTP) signature found in tumor-free adult mice exposed to FLASH (Kunz et al, in rev). The concomitant upregulation of mitochondrial and metabolic genes such as *Dnm1l, Ckmt2,* and *Plin5*^28, 30^ indicates reinforced bioenergetic capacity, while enrichment of genes involved in axonal transport (*Bicd1) 37,* vesicle trafficking in the brain (*Vps26b, Gak*) ^38, 39^, cytoskeletal remodeling supporting axonal growth (*Dock3)* ^40^, and dendritic spine morphology *(Arpin*) ^41^ suggest structural adaptability. Upregulation of genes involved in transcriptional reprogramming in neurons (*Klf3, Klf15)* ^42, 43^ and *Dpy30* known to regulate epigenetic landscape in neural progenitors ^44^ suggest a sustained activity-dependent gene regulation. In parallel, astrocytes of CA3 showed upregulated genes involved in oxidative phosphorylation, cytoskeletal remodeling, membrane trafficking, and immune-related signaling, indicative of a metabolically active, synapse-engaged, and dynamically responsive glial state. Enhanced metabolic fitness, including mitochondrial respiratory chain components (*Cox6a1, Cox8a, Ndufs6, Atp5a1*), antioxidant defense via Sod2, and energy metabolism genes such as *Pygb* and *Arg2* were found. This metabolic reinforcement was paralleled by enrichment of synapse–glia communication pathways, including glutamatergic signaling (*Gria1, Grik4*), GABAergic components (*Gabarap, Glrb*), GPCR-mediated neuromodulation (*Gpr158, Chrm4, Cnr2*), and synaptic scaffolding/vesicle dynamics genes such as *Bsn, Vamp2, Sv2b, and Sh3gl2*, suggesting preserved excitatory–inhibitory balance and altogether promote an efficient neurotransmission as reviewed in^45^. Interestingly, no evidence of a broad pro-inflammatory cascade was found, indeed the upregulation of *Ccl2* and *Alox5*, Fc receptor signaling (*Fcgr1*), and complement-related regulators (*Cd300lb*), rather indicate controlled immune reaction. Finally, the upregulation of chromatin modifiers including *Hdac2, Ash2l, Rnf2* and activity-linked transcription factors such as *Elk4, Fosl1,* and *Hey1* supports sustained transcriptional reprogramming that has been shown to support neuronal plasticity and cognitive functions^46^.

In the neurons of the CA1, the signatures shifted toward synaptic output regulation, intracellular signaling cascades, and transcriptional control pathways, possibly associated with memory consolidation-related plasticity. This is consistent with previous spatial transcriptomic profiling, where memory consolidation-related transcriptional programs in CA1 neurons were predicting and functionally modulating long-term memory formation ^47^. Several anti-inflammatory genes were also found, including *Entpd1*/*CD39,* a phosphatase known to dampen extracellular ATP signaling and therefore limiting inflammation ^48^ and *Cnr2,* a cannabinoid type 2 receptor known to modulate neuronal excitability with potential anti-inflammatory action ^49^, as well as ion channels and transporters (*Asic4, Slc6a5, Slc30a2, Slc9b2)* involved in the maintenance of ionic balance^50^. Strikingly, CA1 astrocytes displayed a robust extracellular matrix, interferon/immune, lipid–steroid metabolic, and chromatin remodeling profile, consistent with structural stabilization and long-term modulation of the synaptic milieu at the principal hippocampal output node.

Together, these findings support a model in which FLASH does not induce a uniform activation program but rather drives circuit-tailored neuron–astrocyte state transitions, as described by Vanrobaeys upon learning tasks in young mice ^47^. Regulatory changes induced in the DG would favor plasticity initiation, in the CA3 enhance recurrent network support and metabolic resilience, and in the CA1 promote structural consolidation and immune-modulated synaptic refinement. This coordinated, region-specific reprogramming suggests that FLASH engages distributed neuron–astrocyte partnerships to reshape hippocampal circuit function at multiple hierarchical levels leading to long-term cognitive preservation.

Our findings provide important translational insights suggesting that FLASH does not simply reduce damage but appears to induce qualitatively different neurobiological programs, including region-specific hippocampal plasticity signatures and a microglial clearance program in the TME, leading to reduced cognitive impairment and complete tumor response. They further suggest that hypo-fractionated radiotherapy regimens may represent a clinically attractive strategy to reduce the logistical and psychological burden associated with multiple treatment sessions, particularly in pediatric and frail patient populations. Nevertheless, several important limitations must be acknowledged before clinical translation can be envisioned. First, the present study employed an intermediate-energy electron FLASH irradiator. The limited penetration of this system precludes treatment of deep-seated lesions, including most primary brain tumors, thereby restricting immediate applicability. Second, the ultra-high dose-rate delivered (> 10^6^ Gy/s) exceeds that currently achievable in most clinical proton or photon FLASH platforms (~100 Gy/s). Whether comparable biological and neuroprotective effects can be reproduced at lower dose-rates and with different radiation qualities remains to be rigorously validated. Third, whole-brain irradiation was used due to the absence of conformal delivery capabilities. Because modern neuro-oncology relies on highly conformal, image-guided approaches to spare healthy tissue, the development and testing of conformal FLASH systems will be essential for meaningful clinical transfer. Finally, although cognitive outcomes after FLASH were improved relative to CONV, performance did not reach the levels previously observed in tumor-free immunocompetent juvenile models ^10, 14^ and is perhaps related to the immunocompromised status used in this study. However, the radiotherapy regimen was composed of 3 fractions of 10 Gy, and we demonstrated that it was ablative; treatment de-escalation using 3 fractions of 9 Gy is feasible and may further enhance the FLASH sparing effect while preserving antitumor efficacy.

In conclusion, while technical and translational challenges remain, the present data support the concept that FLASH irradiation induces qualitatively distinct biological responses that may promote medulloblastoma clearance and mitigate radiation-induced neurotoxicity. Optimization of dose, fractionation, radiation modality, and conformal delivery will be the critical next steps to fully harness the therapeutic window of FLASH radiotherapy and translate its potential cognitive-sparing benefits to children with brain tumors.

## Supplementary Material

Supplement 1

Supplement 2

## Data Availability

All scripts used for the analysis and plotting can be found at https://gitlab.unige.ch/lirr/dsp-pediatric-mb, as well as the raw sequencing data, which is available at https://www.ncbi.nlm.nih.gov/geo/ under accession number pending.

## Key Points:

- Complete response of medulloblastoma and reduction of neurotoxicity with hypo-fractionated FLASH regimen.
- Clearance-prone phagocytic and proteolytic activity in the microglia of the TME.
- Neuron/astrocyte transcriptional crosstalk in the hippocampus.

## Importance of the study :

This study constitutes a milestone for the future implementation of FLASH-RT in the treatment of children with brain cancer. It shows that FLASH does not protect medulloblastoma and on the contrary can be ablative when delivered in 3 fractions of 10 Gy. FLASH promotes a metabolically active, phagocytosis-prone phenotype in microglial cells consistent with immune activation and tumor surveillance, in contrast to the proliferative and immunosuppressive signaling programs induced by CONV. It also shows how FLASH may differentially shape long-term brain function in patients with brain tumors by modifying the transcriptional program of hippocampal subregions known to be critical for memory encoding, pattern separation, and consolidation. In summary, this study supports the idea that FLASH has the potential to shift treatment paradigms and change the dismal therapeutic outcome in patients with brain cancer.
