# Supplementary material for: Early microglial activation in the TME enables FLASH-RT to eradicate medulloblastoma while promoting neuron-astrocyte crosstalk to minimize toxicity in the hippocampus
Source: bioRxiv. 2026 Mar 18:2026.03.16.712103. Preprint. [Version 1] doi: 10.64898/2026.03.16.712103 (PMC13015341; doi:10.64898/2026.03.16.712103)
Supplement: Supplement 1 [file media-1.docx]

**Early microglial activation in the TME enables FLASH-RT to eradicate medulloblastoma while promoting neuron-astrocyte crosstalk to minimize toxicity in the hippocampus**

Michèle Knol, Javier Franco-Pérez et al.

## **Supplementary Material and method**

## **GeoMx Digital Spatial Profiling at early time point.**

Brain samples were fixed in 4% PFA and embedded in paraffin. 5 µm sagittal sections were placed on a slide with one CTRL, one CONV, one FLASH per slide. Spatial transcriptomics was performed according to the standard nanoString GeoMx Digital Spatial Profiler (DSP) protocol^1^. Specific markers were used to identify different cell types : neurons (NeuN, 1:100, Millipore ABN78, with a secondary goat anti-rabbit Alexa 594, 1:1000, Abcam ab150080), astrocytes (GFAP Alexa 532, 1:100, Novus NBP2-3318), and microglia (CD68 Alexa 647, 1:200, Abcam ab305214), as well as a nuclear marker (Syto83, Invitrogen 511364).

## ***Bioinformatics***

Quality control, pre-processing, and further analysis of the data was performed in R (v4.4.0) as per the recommendations from nanoString^2^, using custom scripts and publicly available packages, apart from the normalization of the data, which was done using the Trimmed Mean of M-values (TMM) method. Out of the 110 initial segments, 4 were removed during the quality control because of a low number of nuclei (<50) or aligned reads (<75%), 17 were removed during the detection rate filtering as fewer than 5% of the genes had an expression above the limit of quantification. In summary, quantification from 89 robust segments was possible for downstream analysis. Specificity of the cell type annotations was evaluated by a marker gene expression heatmap, showing concordance with known cell type-associated markers (Supp. Fig. 1A). To assess the sensitivity and coverage of the sequencing, gene detection rates were examined across all cell types and regions, showing acceptable detection rates in all cell types and regions, with higher detection rates in the neurons and the cerebellum (Supp. Fig. 1B). To evaluate possible batch-driven errors, dimensionality reduction using a uniform manifold approximation and projection (UMAP) was performed and did not reveal any clustering (Supp. Fig. 1C and 1D). Together, these analyses confirmed data suitability for downstream analyses.

**Evaluation of neuroinflammation at endpoint.**

Six mice of each group sampled six months after radiotherapy by intracardiac perfusion with PBS and brain was processed to analyze neuroinflammation.

***Total RNA*** was isolated from longitudinal sections of PFA-fixed and paraffin-embedded brain tissues using the MagMAX™ FFPE DNA/RNA Ultra Kit (Applied Biosystems, USA) (n=4 per group). The concentration of RNA isolated was measured using a NanoDrop spectrophotometer (Thermo Fisher, USA). 0.5 μg of RNA from each sample was used to synthesize cDNA using the SuperScript™ VILO™ cDNA Synthesis Kit (Invitrogen, USA). Quantitative real-time polymerase chain reaction (RT-qPCR) was performed using TaqMan™ Fast Advanced Master Mix and different TaqMan® Gene Expression Assays (Applied Biosystems, USA). All genes were assessed in duplicate and our assays contained Template Controls as negative controls containing all the components of the PCR master mix but without cDNA, which was substituted with an equivalent volume of RNase free water. We examined changes in the expression of the inflammatory markers *Il-1b, Tnf-a, Il-6, Nlrp3, Il-4* and *Gsdmd.* Gene expression levels were normalized to endogenous controls *Hprt* and *Gapdh* and calculated using the 2−ΔΔCt method. Assays ID used are listed in Supp Table 1.

***Immunofluorescence*** was used to evaluate microglial activation and astrogliosis in the cerebellum and hippocampus (n=4 per group). Primary antibodies were as follows: rat anti-Iba1 (1:200, ab283346, Abcam, England), rabbit anti-CD68 (1:200, ab283654, Abcam, England), rabbit anti-GFAP (1:200, PA5-16291, Invitrogen, USA). Secondary antibodies were Alexa Fluor 594 goat anti-rabbit IgG (1:500, 111-585-144, Jackson ImmunoResearch, USA) or Alexa Fluor 488 goat anti-rat IgG (1:500, 112-545-16, Jackson ImmunoResearch, USA). Nuclei were visualized by incubating with DAPI. Images were acquired on a Nikon A1R+ inverted confocal microscope using NIS Elements C software and ImageJ analysis (% area positive).

Results were analyzed using a one-way ANOVA and Tukey's post hoc test for multiple comparisons between treatments.

**Supplementary Table.**

**Supplementary Table 1.** Taqman probe details (Applied Biosystems, USA) used for RT-qPCR.

| Target gene | NCBI Reference  Sequence | Taqman Assay ID | Product size |
| --- | --- | --- | --- |
| *Il-1b* | NM_008361.3 | Mm00434228_m1 | 90 |
| *Tnf-a* | NM_001278601.1 | Mm00443258_m1 | 81 |
| *Il-6* | NM_031168.1 | Mm00446190_m1 | 78 |
| *Nlrp3* | NM_145827.3 | Mm00840904_m1 | 84 |
| *Il-4* | NM_021283.2 | Mm00445259_m1 | 79 |
| *Gsdmd* | NM_026960.4 | Mm00509958_m1 | 94 |
| *Hprt* | NM_013556.2 | Mm03024075_m1 | 131 |
| *Gapdh* | NM_001289726.1 | Mm99999915_g1 | 107 |

**Supplementary Figure**


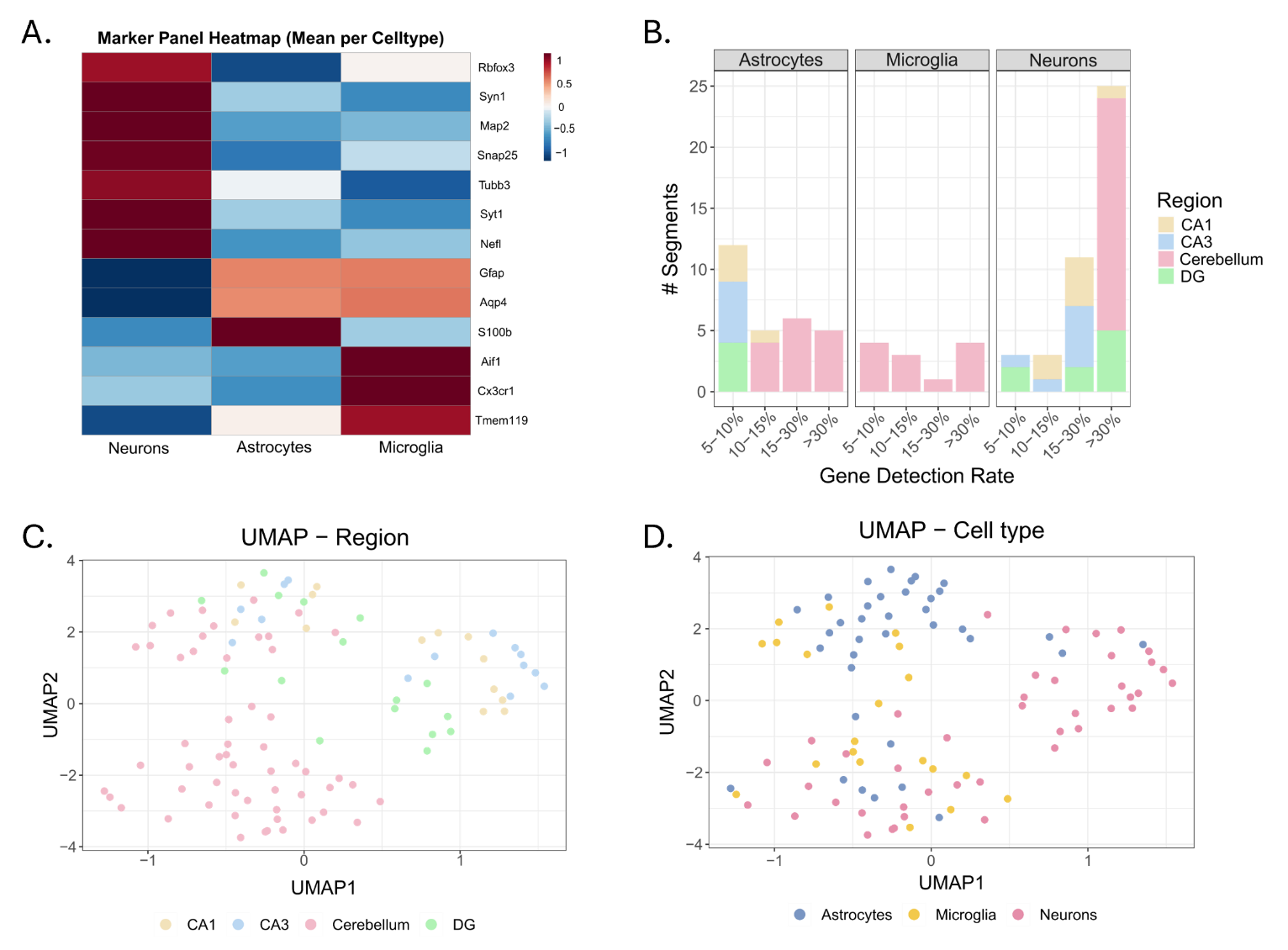


**Supplementary Figure 1. (A)** A heatmap showing the expression of a marker panel per cell type. Normalized expression values per cell type were summed over all regions and normalized per row to obtain expression values between -1 and 1. **(B)** Stacked barplot showing the gene detection rate per region and cell type. **(C)** UMAP dimensionality reduction plot showing all segments (n = 89), colored by region. **(D)** UMAP dimensionality reduction plot showing all segments (n = 89), colored by cell type.
